# A Systematic, Updated Review on the Antidepressant Agomelatine Focusing on its Melatonergic Modulation

**DOI:** 10.2174/157015910792246227

**Published:** 2010-09

**Authors:** Michele Fornaro, Davide Prestia, Salvatore Colicchio, Giulio Perugi

**Affiliations:** 1 Department of Psychiatry, University of Genova, Genoa, Italy; 2 Department of Neurosciences, Catholic University, Rome, Italy; 3 Department of Psychiatry, University of Pisa, Pisa, Italy; 4 Director of the Behavioral Sciences Institute “G. De Lisio”, Pisa, Italy

**Keywords:** Agomelatine, melatonin, depression, mood, cognition, Alzheimer, bipolar disorder.

## Abstract

**Objective:**

To present an updated, comprehensive review on clinical and pre-clinical studies on agomelatine.

**Method:**

A MEDLINE, Psycinfo and Web of Science search (1966-May 2009) was performed using the following keywords: agomelatine, melatonin, S20098, efficacy, safety, adverse effect, pharmacokinetic, pharmacodynamic, major depressive disorder, bipolar disorder, Seasonal Affective Disorder (SAD), Alzheimer, ADHD, Generalized Anxiety Disorder (GAD), Panic Disorder (PD), Obsessive-Compulsive Disorder (OCD), anxiety disorders and mood disorder.

**Study collection and data extraction:**

All articles in English identified by the data sources were evaluated. Randomized, controlled clinical trials involving humans were prioritized in the review. The physiological bases of melatonergic transmission were also examined to deepen the clinical comprehension of agomelatine’ melatonergic modulation.

**Data synthesis:**

Agomelatine, a melatonergic analogue drug acting as MT_1_/MT_2_ agonist and 5-HT_2C_ antagonist, has been reported to be an effective antidepressant therapy.

**Conclusions:**

Although a bias in properly assessing the “sleep core” of depression may still exist with current screening instruments, therefore making difficult to compare agomelatine’ efficacy to other antidepressant ones, comparative studies showed agomelatine to be an intriguing option for depression and, potentially, for other therapeutic targets as well.

## INTRODUCTION

The definition of depression essentially relies on depressed mood. Yet, depression represents a multi-dimensional condition. Among others, psychomotor symptoms, sleep disturbances, somatic, pain symptoms, anxiety, diurnal variation and seasonal patterns, could lead to very heterogeneous pictures, possibly underpined by different pathogenetic mechanism [1]. Hence, the associate burden may vary as well. Furthermore, Mood Disorders (MDs), particularly Major Depressive Disorder (MDD), are spread among general population, both with sub- and full-threshold manifestations: MDD has been estimated to be the fourth major cause of disability worldwide, and may become second only to cardiovascular diseases by around 2020 [2]. Although the social relevance of the phenomenon, less than 50% of all patients treated with the currently available antidepressants show full remission [3], with a large number of subjects showing residual and relapsing symptoms [4] and general poor antidepressant outcome still in a too high number of cases as demonstrated by the Sequenced Treatment Alternatives to Relive Depression (STAR*D), the largest antidepressant trial ever [5, 6]. A main reason for this is the still incomplete knowledge about the pathogenetic mechanisms of depression and about the inner actions of available antidepressants [3]. Also, a non-dimensional, categorical approach [7] may lead to the use of a single diagnosis to include heterogeneous clinical pictures, therefore leading to a non patient-tailored psychopharmacotherapy. Additionally, even when effectiveness is well-documented, current anti-depressants may be associated with impairing side-effects which may account for most of the discontinuation cases [8].

These considerations prompt for a better recognition and for an adequate clinical management of depression but also for the development of more selective and new-targeting drugs. 

First Generation Antidepressants (FGAs) include Monoamine Oxidase Inhibitors (MAOIs) and Tricyclic Antidepressants (TCAs, 60s), while Second Generation Antidepressants (SGAs, 80s-90s) include Selective Serotonin Reuptake Inhibitors (SSRIs), Norepinephrine and Serotonin Reuptake Inhibitors (SNRIs), Norepinephrine Reuptake Inhibitors (NARIs), Norepinephrine and Specific Serotonergic Antidepressants (NASSAs) and Serotonin 5-HT_2A _Antagonists/Reuptake Inhibitors (SARIs) [3]. SSRIs still represent the most currently prescribed antidepressant class of drugs, nevertheless their efficacy has been questioned [9-16].

Both the FGAs and SGAs are monoamine-based antidepressants. New monoamine-based antidepressants are currently in development or marketed, and they include 5-HT_4_ and 5-HT_6_ agonists as well as 5-HT_7 _antagonists [3]. According to the monoamine hypothesis of depression, monoamine-based antidepressants have been hypothesized to increase the synaptic availability of monoamines (which could be decreased in course of depression). Yet, this hypothesis of depression has been criticized since it was evident that increased availability of monoamines induced by antidepressants develops in a matter of hours with the therapeutic effect onset only after a mean lag-phase of several weeks [17]. Therefore, in the following decades, new non-monoamine-based antidepressants have been studied. They include the NK-1 receptors antagonists, CRF1 antagonists and the glutamatergic agents (NMDA blockers, AMPAkines, mGlu modulators, riluzole, lamotrigine) [3].

## AGOMELATINE: A NOVEL APPROACH TO DEPRESSION

Recently, a novel approach to depression, focusing on circadian rhythms, has been the basis for the development of agomelatine (*N*-[2-(7-methoxy-1-naphthyl)ethyl]acetamide or S-20098), a melatonin (MT) analogue drug with a entirely new mechanism of action [18].

In mammals, changes in the sleep-wake cycle and in the periodicity of circadian rhythm profoundly influence the state of mood, which they have already been proposed as candidate markers [19, 20]. Likewise, it has been shown that manipulations of circadian rhythms, such as a total of REM sleep deprivation or phase advance in the sleep-wake cycle, may have antidepressant action. This has also been considered the rational for complementary or alternative strategies such as the “sleep deprivation therapy” and similar approaches [21]. Anyway, since studies demonstrated possible persistent sleep changes in the remission phase of depression, it is unclear whether it is a causative factor or part of the clinical picture [22]. Possibly, this may be also due to an incomplete action of antidepressant therapy, since currently available drugs may be unable to address the sleep depressive symptomatology. To date, agomelatine, represents the only available MT_1_/MT_2_ melatonergic receptors agonist and 5-HT_2C_ antagonist (SAR), shown to induce resynchronization of circadian rhythms and antidepressant action in humans, [23, 24]. By avoiding 5-HT_2A_ stimulation, agomelatine shows a more favorable side-effect profile compared SSRIs, concerning sexual functioning, weight-gain (drugs blocking the 5-HT2C and histamine receptors are usually associated with weight-gain although 5-HT2C per se shouldn’t be necessarily associated with this effect) and Gastro-Intestinal (GI) disturbances, without exhibiting discontinuation symptoms [11, 18, 25-27].

## METHOD

To identify relevant articles for this review, searches of the online databases MEDLINE and EMBASE were conducted using combinations of the search terms “agomelatine”, “melatonin”, “S20098”, “antidepressant”, “efficacy”, “safety”, “pharmacokinetic”, “pharmacodynamic”, “receptor binding”, “depression”, “Major Depressive Disorder” (MDD), “Bipolar Disorder” (BPD), “Seasonal Affective Disorder” (SAD), “Attention Deficit Hyperactivity Disorder” (ADHD) and “anxiety disorders”. Additional articles were identified scanning the reference lists of the retrieved articles. All English-language articles reporting original data related to agomelatine for major depression were included in this review while just RCT, non antidepressant-related agomelatine references were prioritized. Most relevant pre-clinical and clinical studies about agomelatine have been reported in Tables **1**, **2** respectively.

## PHARMACOKINETICS

In humans, agomelatine is well absorbed following oral administration, but absolute bioavailability is relatively low (about 5-10%) due to its high first-pass metabolism [28], which may be considered in special populations such as the elderly or hepatic disordered patients. When given as a single 25- or 50mg amount, blood concentrations increased more than proportionately to the dose, possibly due to saturation of first-pass metabolism. Agomelatine has also moderate distribution in humans, with a volume of distribution of approximately 35 L., and is 85-95% bound to plasma proteins (again, this could taken in account prior prescription in special populations) [29]. Agomelatine appears to be extensively metabolized by the cytochrome P450 isoforms 1A1, 1A2 and 2C9 (majority of psychiatric medications undergo 2D6 or 3A4 or 1A2) to hydroxyl, desmethyl and epoxide metabolites with less activity than the parent drug. A major oxidative metabolite in humans, 3-hydroxy-7-desmethyl-agomelatine, has low affinity for MT_1_, MT_2_ and 5-HT_2C_ receptors. The drug is eliminated mostly by urinary excretion of the metabolites (61-81% of dose in humans), with a small amount of the diol metabolite undergoing fecal excretion; the mean terminal elimination half-life is 2.3 hours [30].****

## PHARMACODYNAMICS

Agomelatine acts as a MT_1_ and MT_2_ agonist (reported to act as a partial agonist on the MT receptors in the pars tuberalis of the rat) [31] and as a 5-HT_2C _and 5-HT_2B_ serotonin (5-hydroxytryptamine, 5-HT) antagonist [32]. Blockade of 5-HT_2C_ receptor, a subtype of 5-HT that binds the endogenous 5-HT neurotransmitter being a G_q_/G_11_ protein-coupled receptor (GPCR) mediating excitatory neurotransmission [33], causes release of both Norepinephrine (NE) and Dopamine (DA) at the frontocortical dopaminergica and adrenergic pathways [32] by different classes of drugs including the SSRI fluoxetine and norquetiapine, the principal metabolite of the atypical antipsychotic quetiapine [34, 35].This is why these agents could be called Norepinephrine and Dopamine Disinhibitors (NDDIs) as coined by Millan (2003) [36], acting across the peripheral and brain Central Nervous System (CNS) including the striatum, prefrontal cortex, nucleus accumbens, hippocampus, hypothalamus, amygdala, and many other areas [37]. The profile of pharmacological actions predicts not only antidepressant actions due to the NDDI mechanism of 5-HT_2C_ antagonism [38] (interestingly, studies on 5-HT agonists as potential antidepressant drugs were discontinued within the recent years [39]),but also sleep-enhancing properties due to MT_1 _and MT_2_ potent agonist actions [32]. The expression of MT_1_ receptors has been shown to have diurnal rhythmicity, regulated by light and the internal clock, whereas the expression for mRNA of 5-HT_2C_, but not 5-HT_1A_ or 5-HT_2A_ receptors, has a circadian rhythm pattern in mammals [40].

While functional desensitization of 5-HT_1A_ auto-receptors in the Dorsal Raphe nucleus (DRn) occurs after chronic administration of several classes of antidepressants and it is considered as a core mechanism implicated in the mood restoration, neither the acute or chronic treatment with agomelatine changed the density of 5-HT_1A_ receptors and their coupling with G proteins in the DRn and the hippocampus in rats nor in the Frontal Cortex (FC) [41]. These data indicate that the antidepressant action of agomelatine is not mediated by the same mechanisms of SSRIs and TCAs [41, 42].

Also, the DA-ergic transmission may be indirectly modulated by the melatonergic one; starting with light-stimulation at the Pigmented Epithelium (PE) of the retina, hosting D_2_-like receptors. A balance between GABA, DA and MT exists all over the CNS [43-47] as demonstrated by Electroretinographic (ERG) studies both in health volunteers [48] and in course of SAD [49]. Agomelatine’ “emotional blunting”, due to DA direct antagonism, should therefore be hypothesized. However, microdialysis studies reported dopamine levels in the nucleus Accumbens (ACn), a structure proven to be involved in course of depression in rats [50] and the striatum to be unaffected by agomelatine [51] whereas it remarkably rises at the prefrontal cortex of rats [52, 53]. The 5-HT_2C _blockade also enhances the activity of FC’s DA-ergic and adrenergic activity, while a stimulatory and entraining effect of melatonin (and agomelatine) on Tubero-Infundibular DA-ergic neurons (TIDA) activity and inhibition of Prolactin (PRL) secretion, seems to be independent on 5-HT_2C_ blockade [32, 54].

On a solely chronobiological basis, agomelatine should not behave differently from an agent like ramelteon (another MT_1_/MT_2_ agonist unaffecting 5-HT_2C_ neurotransmission). Agomelatine, on contrast, has a dual phased action: at night, its sleep-promoting melatonergic effects prevail over its potentially anti-hypnotic 5-HT_2C_ blockade, whereas during the day, its antidepressant action *via *5-HT_2C_ inhibition is uncoupled from melatonin’s nocturnal actions (this may also be considered as an advantage of agomelatine vs. other classes of antidepressants) [19].

## The MT_1_/MT_2_ MODULATION. THE PHYSIOLOGICAL MELATONERGIC BASES OF AGOMELATINE

Agomelatine and melatonin are not synonymous neither the supposed antidepressant MT_1_ and MT_2_ agonism should be considered in the strict sense of hormone substitutive modulation. On the other hand, agomelatine, next to the exogenous hormone, is the most melatonin-mimic agent developed for antidepressant therapy [55].

There are three types of receptors for melatonin: 1 and 2, which are both involved in sleep, and 3, which is actually the enzyme NRH: quinine oxidoreductase 2 and not thought to be involved in sleep physiology. There are several different agents acting at melatonin receptor sites. Melatonin itself, available over the counter, acts at melatonin 1 and 2 receptors as well as at the melatonin 3 side. Ramelteon seems to provide sleep onset though not necessarily sleep maintenance, being ineffective for jet lag treating [56-58], in contrast to the MT1/MT2 agonist tasimelteon [59], without modulation of the 5-HT-ergic transmission.

In order to assess the clinical actions of agomelatine, the melatonergic modulation and serotonergic one should considered separately, briefly recalling the physiologic mechanisms of the hormone melatonin and, when comparative study have been performed, reporting a side-by-side profile of both.

### Synthesis of Melatonin

The indoleamine melatonin (N-acetyl-5-methoxytrypt-amine) is synthesized from the amino acid tryptophan *via *5-HT synthesis. Production of melatonin by the pineal gland is under the influence of the Suprachiasmatic Nucleus (SCN) of the hypothalamus, which receives information from the retina about the daily pattern of light and darkness. Both SCN rhythmicity and melatonin production are affected by non-image-forming light information traveling through the retino-hypothalamic tract (RHT). The melatonin signal forms part of the system that regulates the circadian cycle by chemically causing drowsiness and lowering the body temperature, but is the SCN that controls the daily cycle in most components of the paracrine and the endocrine system rather than the melatonin signal. The responsiveness of the SCN to melatonin is therefore strongly regulated by the circadian clock, while chronic melatonin (or agomelatine) agonism of the SCN melatonin receptors didn’t result in their desensitization in animal models [60]. Also, both melatonin and agomelatine activities on circadian rhythms depends on the SCN integrity but not the pineal gland as demonstrated in animal studies [61-63] in a dose-dependent fashion [64].

### Aging and Melatonin

Under the age of 3 months, little melatonin is secreted, and there is no variation with light exposure. The production peak is reached at the age of 3 years and then this declines, especially during puberty, to a level which is maintained until around age of 40 before it fall further [65, 66]. This may also account for a variety of depression-related patterns and outcomes among different aged populations. MT and its receptor agonists, including agomelatine, correct age-related changes in circadian response to environmental stimuli in rodents, and could prove to be useful in treating/preventing or delaying disturbances of circadian rhythmicity and/or sleep in older people [67, 68].

### Light and Circadian “Rhythms”

Production of melatonin by the pineal gland is inhibited by light and permitted by darkness. Hence melatonin has been called "the hormone of darkness" and its onset each evening is called the Dim-Light Melatonin Onset (DLMO). Secretion of melatonin, as well as its level in blood, peaks in the middle of the night, and gradually falls during the second half of the night, with normal variations in timing according to an individual's chronotype [69-71]. This justifies the use of melatonin, and its analogue agomelatine, to promote sleep in those with delayed sleep onset or to reset the internal clock that occurs with jet lag, shift working, or due to other causes [56, 72-74], possibly recovering from the Delayed Sleep-Phase Syndrome (DSPS) too [75]. Light-time exposure (“photoperiod”) is a pivotal element in regulating circadian rhythms, thus it is unsurprising that bright “light therapy” stimulation has already been proposed as an antidepressant and SAD treatment option [19, 76, 77].

### SCN and the Anxiolytic Effect

Melatonin appears to have two effects on the SCN which are mediated either by a direct effect on the circadian rhythm generating cells or by activation by the GABA-ergic neurons within the SCN which inhibits its activity [78-80]. A GABA-ergic modulating action, along with a 5-HT_2C_ one, may also account for the reported anxiolytic effects of agomelatine [81-83]. While melatonin-like drugs have been reported to overlap the GABA agonists activity (such as diazepam), neither agomelatine nor melatonin substituted benzodiazepines in anxiety-stressed rats trained to discriminate the different drugs, suggesting that agomelatine anti-anxiety effect may be not as addictive as the diazepam’ one (therefore being a core feature when it comes to choice the proper drug in abusers and other addictive-behavior populations) [83-86]. On the other hand, agomelatine’ anxiolytic effect strictly resembles those of selective 5-HT_2C_ antagonists, thus the melatonergic-agonism may be not entirely account for [25]. Efficacy of agomelatine in GAD has been reported by an RCT investigation by Stein *et al*. (2008) but further investigations in PD, OCD, and other Anxiety Spectrum disorders are needed [87].

### Cognitive Functions, Alzheimer’s Disease and Delirium

Melatonin receptors appear to be important in mechanisms of learning and memory in mice [88] and the hormone can alter electrophysiological processes associated with memory, such as Long-Term Potentiation (LTP). Spatial visual memory, as well as the ventral hippocampal expression of the synaptic Neural Cell Adhesion Molecule (NCAM), were reported to improve in animal model treated with agomelatine [89].

The first published evidence that melatonin may be useful in Alzheimer's disease was the demonstration that this neurohormone prevents neuronal death caused by exposure to the amyloid beta protein, a neurotoxic substance that accumulates in the brains of affected patients [90, 91]. Melatonin also inhibits the aggregation of the amyloid beta protein into neurotoxic micro-aggregates which seem to underlie the neurotoxicity of this protein, causing neuronal death and formation of neurofibrillary tangles, which are the other neuropathological landmark of Alzheimer's disease [90]. Melatonin has been shown to prevent the hyperphosphorylation of the tau protein in rats [92]. Hyper-phosphorylation of tau protein can also result in the formation of neurofibrillary tangles [92]. Studies in rats suggest that melatonin may be effective for treating Alzheimer's disease [92]. These same neurofibrillary tangles can be found in the hypothalamus in patients with Alzheimer's disease, adversely affecting their bodies' production of melatonin. Patients with this specific affliction often show heightened afternoon agitation, called “*sundowning”*, mainly due to cholinergic transmission. This phenomenon, has been shown in many studies to be effectively treated with melatonin supplements taken at bedtime [92]. The sundowning syndrome (possibly also related to progressive light-decrease) often characterizes many delirium cases and mood and/or cognitive disorders (especially when essentially due to cholinergic hypo-functioning). Indeed, melatonergic agonism could indirectly reduce the DA-ergic central activity and may promote the GABA-ergic activity (therefore complicating the delirium and other cognitive impairments pictures). While melatonergic drugs could be considered in recovering a better profile light-rhythm, they could carefully considered prior being administered to cognitive-impaired patients both in course of neurodegenerative diseases or when cognitive symptoms occur in course of depression as part of the illness or as potential consequences of some antidepressant therapies, as it may occur with long-term treatments with SSRIs drugs.

### Melatonin and Dopamine-Related Motor Disorders

Inhibition of DA release by melatonin has been demonstrated in specific areas of the mammalian CNS (especially, the hypothalamus, hippocampus, medulla-pons and retina) [93]. Anti-DA-ergic activities of MT, mediated by BDZ/GABA_A_ receptors, has also been demonstrated in the striatum [94]. DA-ergic transmission has a pivotal role in the circadian entrainment of the fetus, in coordination of body movement and reproduction; MT may also modulate DA-ergic pathways involved in movement disorders in humans. In Parkinson patients, MT may exacerbate symptoms (because of its putative interference with DA release) and, on the other side, protect against neurodegeneration (by virtue of its antioxidant properties and its effects on mitochondrial activity). MT appears to be effective in the treatment of Tardive Dyskinesia (TD), a severe movement disorder associated with long-term blockade of the postsynaptic D_2_ receptors induced by antipsychotic drugs (especially by first generation ones). The interaction of MT with the DA-ergic system may play a significant role in the non-photic and photic entrainment of the biological clock [95] as well as in the fine-tuning of motor coordination in the striatum [93]. These interactions and the antioxidant nature of melatonin, including degenerative and possibly primary cases of rethinopathies due to antipsychotic treatment [96], may also suggest agomelatine and other melatonergic-drugs to be considered as potentially helpful in the treatment of DA-related disorders, which could also be taken into account in case of motor side effects eventually due to some antidepressant therapies as it is the case of potential extrapyramidal effects of SSRIs antidepressants [97].

## MELATONIN, AGOMELATINE AND NEUROPLASTICITY: PRE-CLINICAL, ANIMAL MODEL AND CLINICAL FINDINGS

Among the biological bases of depression, an impairment of neuroplasticity and cellular resilience has been proposed [98] with antidepressant medication reported contributing in its normalization [99-101]. Chronic stress, excess of concentrations of glutamate, biogenic amines and glucocorticoids affect the morphology of hippocampal CA3 pyramidal neurons, resulting in a pronounced debranching of apical dendrites. This affect can be blocked or counteracted by different compounds including antidepressant drugs.

A 2008 study by Gressens and colleagues demonstrated the efficacy of agomelatine in neuroprotection and neuroplasticity of newborn rats. White matter cysts (mimic human periventricular leukomalacia), previously induced by intraperitoneal injection of glutamatergic-like agents (ibotenate), partially recovered with melatonin (administered 2h within acute lesion) and agomelatine (up to 8h after) [102]. Neurocognitive and antidepressant actions have also been demonstrated in “depressed” rodents (using the Forced Swimming Test (FST)) comparing agomelatine vs. imipramine or vs. melatonin or vs. fluoxetine [103], while mice circadian system was also proved to improve with agomelatine therapy (using the Phase Response Curve (PRC) record) as long as motor activity initiative (using the daily wheel revolution Chronobiology Kit^®^) [20, 104]. Agomelatine treatment also resulted in prolonged overstimulation of melatonin receptors, thus attenuating the effect of light on the circadian timing system [105].

## MELATONIN, AGOMELATINE AND MOOD: A FASCINATING CONNECTION MEDIATED BY CIRCADIAN RHYTHMS

Profound disturbances in sleep architecture often occur in MDD and Bipolar Disorders (BPDs) [19]. MDD with Melancholic features is associated with sleep-awake phases advance, often showing up with praecox final awake and reduced REM latency. MDD with seasonal patterns (MDDSP) is clinically characterized by depressed mood occurring at almost the same time every year since the disorder is first experienced [106]. US prevalence has been estimated to be at least 5% among general population with F:M ratio of about 5:1 [107]. Since most of the world population is located further from the equator, more people could be affected, prompting for a better comprehension of the phenomenon. The pathophysiology of MDDSP is not fully understood, although it is assumed to be associated with altered circadian rhythms. Basic circadian rhythms are regulated by several endogenous or exogenous pacemakers, which major endogenous one is probably located in the hypothalamus. One of the major exogenous pacemakers is the light–dark cycle, in which different durations of light or dark hours affect the timing of sleep induction, hormone secretion, and many other biological rhythms. In health, euthymic subjects, the ratio of light to dark hours triggers the SCN to induce certain activities, including sleep, hormone secretion, and the secretion of melatonin (which may only serve as a marker associated with changes related to MDDSP) *via *stimulating the pineal gland. MDDSP is characterized, among other things, by a basic state of “phase-delay” circadian rhythm. This means that the same triggered activities (by the SCN) are induced at a later time in the day (24-hour clock) than in non-MDDSP patients. Empirical data suggest that when a person is exposed to bright light during the light hours, the SCN is stimulated to induce its activities at an early time in the 24-hour cycle. This is termed ‘phase-advance’ circadian rhythm. If it is administered to a MDDSP patient, the ‘phase-advance’ is superimposed on a “phase-delay” status, which may bring the system to an equilibrium, normalizing circadian rhythms, and at the same time ameliorating the depressive symptoms of MDDSP [108-110], Fig. (**1**).

### Melatonin, Agomelatine and Bipolar Spectrum Disorders

Melatonin has been considered for BPD, SAD, and other clinical pictures where circadian disturbances are involved [111, 112] as well other stress-related conditions. About 20 years ago Ehlers *et al*. articulated the hypothesis concerning the way in which stressful life events that disrupt an individual’s normal routines (“social zeitgebers”) could initiate a cascade that – in vulnerable subjects – might lead to an episode of depression or mania [113]. Among psychiatric illnesses, BPD is second only to unipolar depression as a cause of global disability [114] and may largely go under-diagnosed [115]. Symptomatic patients with BP-I disorder experience depressive symptoms three to four times more than manic symptoms [116, 117] and symptomatic patients with BP-II disorder experience depressive symptoms approximately 39 times more than hypomanic ones [118]. These considerations prompt for a better recognition and management of depressive states associated with BPD and their clinical features. It has been observed that BPD might have a "trait marker" of hypersensitivity of the melatonin receptors [119]. Anyway, this could be contrasted with drug-free recovered bipolar individuals not showing light hypersensitivity [120]. A comprehensive review by Gao *et al*. (2005) focused on RCT studies of newly introduced drugs (including agomelatine) for the acute and long-term treatment of bipolar depression. Preliminary open-label observations of agomelatine addiction to lithium or valproate in the treatment of bipolar depression showed the efficacy of the melatonergic drug at doses of 25mg/day after 6 weeks of augmentation treatment. Anyway these results need RCT confirmations [121].

The nature of disruption of melatonin secretion in MDD has been under intensive study ever since it has been proposed as “*low melatonin syndrome*” [122] and replicated by a number of studies [19], whereas there is no evidence of agomelatine increasing the levels of melatonin. However, increases in melatonin secretion in depressive symptomatology has also been reported [123]. The differences could be due to changes in depressive symptomatology or to the pattern of melatonin secretion, inasmuch as there are studies showing that daytime melatonin secretion in depressives is increased [19, 124]. Interestingly, lower levels of illumination in post-menopausal women have been reported to be associated with more complaints of sleep and depressive symptoms [125] whereas post-partum depression was already suggested as a possible marker/predictor of bipolar depression [126]. Bright light treatment of women suffering from ante-partum depression advanced the rhythm of melatonin secretion and also mitigated depressive symptoms [19]. Also, a marked reduction in sleep during the night immediately before switching from depression to mania was noted in bipolar depressed patients [127].

Yet, measurement of melatonin levels has shown significantly lower levels in unipolar and bipolar depressed patients [128]. The significance of the association of sleep disturbances and melatonin levels in bipolar depressed patients is still far away from a satisfactory knowledge.

### Melatonin, Agomelatine and Major Depression

Both animal and human studies demonstrated agomelatine to be an effective treatment for MDD [38, 81, 129-132]. The efficacy of agomelatine in severe depression [133, 134] has been investigated by Montgomery and Kasper (2007) by a pooled analysis of 3 positive placebo-controlled studies (doses were 25 to 50mg/die) proving it to be an effective treatment [135]. Sleep abnormalities in depression are mainly characterized by increased Rapid Eye Movements (REM) sleep and reduced Slow-Wave Sleep (SWS) [40] with most of available antidepressants (including TCAs and SSRIs) causing REM sleep suppression and increasing in REM sleep onset latency [136, 137]. Decreased cholinergic activity and increased 5-HT-ergic one are the two main factors affecting REM sleep suppression [40]; the decrease in amount of REM sleep appears to be greatest during the early phases of treatment, gradually diminishing during long-term treatment, except after Monoamine Oxidase Inhibitors (MAO-I) administration when REM sleep is often absent for many months. Many antidepressant medications, including SSRIs, have repeatedly been reported to worse sleep, mainly due to 5-HT_2_ stimulation; on the other hand, excessive sleep, daytime sleepiness and sedation may be experienced by patients tacking antidepressant medications [40]. 5-HT_2_ blocking antidepressants, as mirtazapine, have been shown to improve sleep continuity and may therefore represent a good option for depressed patients with marked insomnia. Agomelatine (25mg/day for 6 weeks) contributes to restore sleep architecture in depressed patients as shown by polysomnography records, improving sleep quality and continuity: SWS’s duration increases without modifying REM sleep time [138]. An RCT investigation by Lemoine *et al*. (2007) compared venlafaxine to agomelatine for subjective sleep in course of MDD showing a greater improvement with the melatonergic drug [139]. A RCT study by Kennedy *et al*. (2008), also investigated the sexual side effect profile of agomelatine in comparison with venlafaxine [140].

## SAFETY AND TOLERABILITY

A review by Ghosh and Hellewell, (2007) evidenced the effect and tolerability of agomelatine in MDD [141] which resulted better tolerated than SSRIs and SNRIs in MDD patients treated for 4-8 weeks on doses ranging from 5 to 100mg/day [142], including a favorable sexual functioning profile which is an important cause of SSRIs non-compliance [143], Table **1**. As reported by Loo *et al*. in a 2003 RCT investigation, agomelatine did not modify cardiovascular parameters, including ECG recordings, neither provoked biological abnormalities, weight or vital signs changes; slightly more adverse effects and severe treatment-related adverse events occurred, however, in the 100mg/day group (i.e. nausea was generally more frequent in the paroxetine comparison group) [142]. RCT studies by Kennedy and Emsley (2006) and Olié and Kasper (2007) also confirmed good tolerability (similar to placebo) of 25-50mg/day of agomelatine [18, 144]. Montgomery *et al*. (2004) focused on discontinuation symptoms: patients abruptly switched from agomelatine 25mg/day (12 weeks) to placebo were compared to those continuing on the same regimen, to placebo-placebo and also compared to paroxetine-to placebo (20mg/day for 12 weeks) vs. paroxetine-paroxetine subjects. After one week, paroxetine discontinued patients experienced significantly more discontinuation symptoms (*P*<0.001), compared to paroxetine-continuing ones. On the other side, 2 weeks after agomelatine cessation, patients discontinuation symptoms were comparable to those of the placebo group [15, 81]. Better acceptability of agomelatine (25 and 50 mg/day) compared with paroxetine (20mg/day) in healthy male volunteers was also assessed by an 8-week, placebo-controlled study by Montejo *et al*. (2008) [145]. A large sampled (subjects=339) 24-week, double-blind, placebo-controlled RCT study by Goodwin *et al*. (2009) demonstrated agomelatine to prevent relapse in patients treated for MDD with no appreciable withdrawal syndrome in comparison to placebo (confirming efficacy seen in short-term studies) [146]. Yet, despite a general good tolerability profile, prescribers should note the requirement to conduct liver function tests (LFTs) in accordance with the recent guidance by the European Agency of Medicines (EMEA) since recent literature evidences prompt for a risk for elevation of liver enzymes with agomelatine (although underpinning mechanism is still under investigation) [147-149]. Finally, a lack of literature data of agomelatine safety and tolerability in older, pregnant or adolescent patients still exists, whereas pharmacokinetic issues suggest prudence in these populations.

## LIMITS

At writing time, a paucity of investigations about agomelatine antidepressant efficacy and tolerability in human samples still exists, although most of available data suggest its efficacy and safety at doses of 25-50mg/kg. In comparison to other antidepressants, the tolerability profile of this agent makes it a treatment option for patients who cannot tolerate currently available antidepressants [150].

Preliminary observations on agomelatine use in depressed subjects are available although its peculiar pharmacodynamic profile suggests to explore also other conditions. The MT_1_ and MT_2_ agonism, as well the 5-HT_2C_ (and 5-HT_2B_) antagonism, involve more complex neuronal firing mechanisms (involving DA-ergic and GABA-ergic modulations), further complicating the comprehension of agomelatine’ biological and clinical actions. Additionally, the Hamilton Rating Scale for Depression (HAM-D) and Anxiety (HAM-A), Clinical Global Impression (CGI) and Montgomery-Asberg Depression Rating Scale (MADRS) showed significant improvement with agomelatine vs. placebo [151, 152]. Possibly, the rating instruments may be inappropriate to adequately assess the sleep and circadian symptoms of depression, leading to a bias in comparative studies involving agomelatine and other classes of antidepressant.****

## CONCLUSIONS

Expectations from new antidepressant therapies go beyond efficacy alone, to include advantages in tolerability and safety. Although current diagnostic instruments and rating scales may be unable to cover the sleep disturbances of depression in a proper and comprehensive manner, agomelatine efficacy on MDD symptoms has been pointed out by preliminary observations both on pre-clinical and clinical samples. Due to its pharmacological profile, agomelatine does not induce the side effects related to common antidepressant prescriptions (i.e. gastrointestinal disorders, weight gain, sexual dysfunction, serotonin syndrome, insomnia, discontinuation syndrome, and others) [153], making the drug an intriguing option in the antidepressants scenario.

## DISCLOSURES

The authors read and approved the final version of the manuscript, having no conflicts of interests nor financial support to state.

## Figures and Tables

**Fig. (1):**
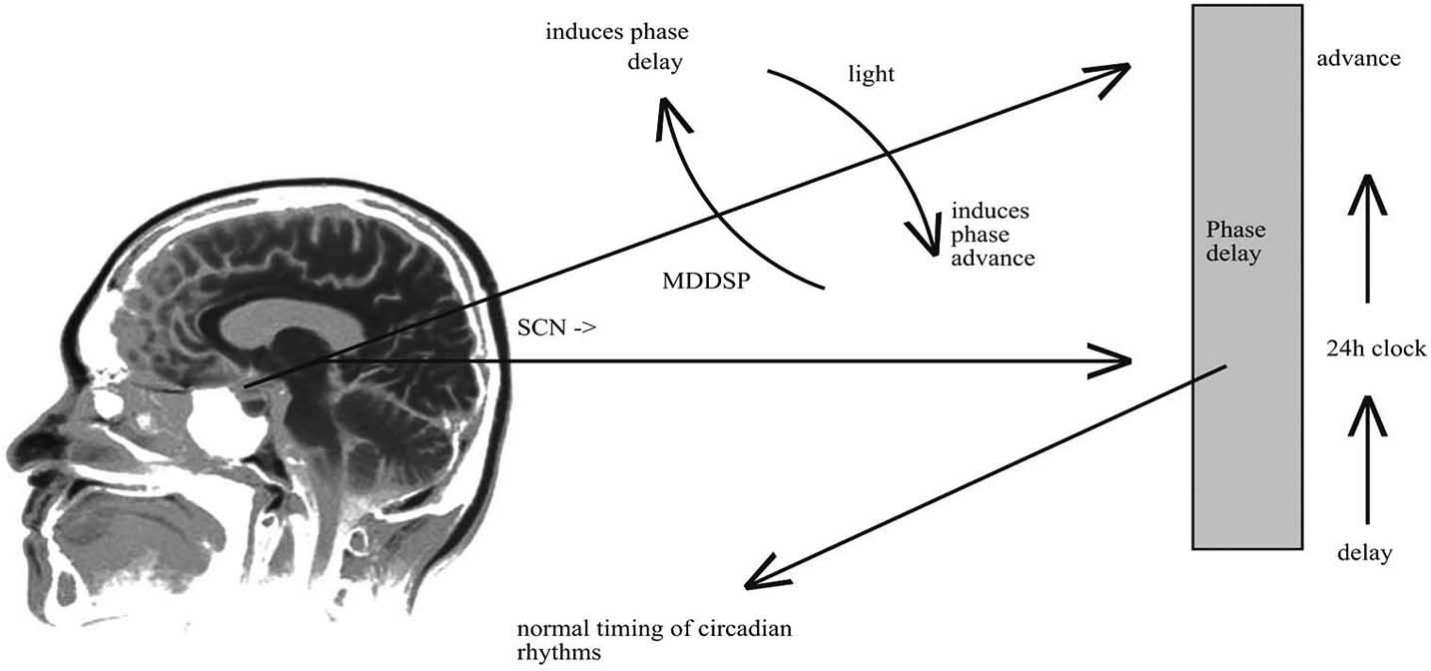
MDDSP and sleep phases switches.

**Table 1:** Agomelatine in Mood and Anxiety Conditions and More. Current Literature Evidences in Pre-Clinical and/or Animal Model Studies

Author, Date	Sample	Condition	Method	N Subjects	Dose/Range	Results
**Table 1 part a **						
Conboy *et al*., (2009)	Animal model (murine)	RAWM stress-induced memory impairment	RAWM stress-model: 4 arms: vehicle-no stress, vehicle-stress, agomelatine-no-stress & agomelatine-stress	20 adult caves, 4 groups (5 subjects each)	Agomelatine 10mg/kg or vehicle 1mg/kg 1% hydroxyethylcellulose	Agomelatine blocked the predator stress-induced impairment of spatial memory. Both stressed and non-stressed agomelatine-treated rats showed an increase in the ventral hyppocampal expression of synaptic Neural Cell Adhesion Molecule (NCAM)
Gressens *et al*. (2008)	Animal model (murine)	Periventricular leukomalacia and cerebral palsy model (induced by ibotenate injections)	Intracerebral injection in newborn mice of ibotenate (glutamate analogue) to develop human periventricular leukomalacia. Melatonin (already shown to have neuroprotective effects in mouse model) was compared to agomelatine to investigate the neuroprotective effects of the latter drug.	Swiss pups (unspecified number)	10µg ibotenate were injected into developing mouse brains, inducing glutamate NMDA and metabotropic receptors activation.	Although agomelatine and melatonin did not prevent the initial appearance of white matter lesions, they did promote secondary lesions repair when given within the first 2h following the excitotoxic insult (up to 8h later for agomelatine)
BertainaAnglade *et al*. (2006)	Animal model (murine)	Animal learned helplessness model	Stress response levels were recorded after pretreatment with agomelatine, melatonin, SB-242084 (selective 5-HT2c antagonist) or agomelatine + S22153 (melatonin receptor antagonist), compared to imipramine	40 (10 per group) wistar rats	Agomelatine 2, 10, 50, 100 mg/kg; melatonin 2, 10, 50 mg/kg; SB-242084 0.31, 1.25, 5, 20 mg/kg; S22153 20 mg/kg; imipramine 64 mg/kg	Pretreatment with agomelatine, as with imipramine, decreased the number of escape failures, reflecting an antidepressant-like properties
Loiseau *et al*. (2006)	Animal model (murine)	Animal model of anxiety	Rats behavior was monitored in the punished drinking test, the safety signal withdrawal operant paradigm, the elevated plus maze and hypophagia-induced novelty, after the administration of agomelatine or melatonin or agomelatine+diazepam or melatonin+diazepam	Unspecified n.	Agomelatine 20-40 mg/kg; melatonin 20-80mg/kg; diazepam 0.25 mg/kg	Agomelatine displayed a pattern of anxiolytic activity on its own (increasing the number of foot shocks received by rats, but non enhancing food consumption in unfamiliar environment); furthermore it potentiated the anxiolytic effects of diazepam; melatonin was less active
Papp *et al*. (2006)	Animal model (murine)	Elevated plus maze, vogel, conditioned footshock-induced ultrasonic vocalization tests.	Monitoring of rats after the morning and evening administration of agomelatine, melatonin, diazepam, buspirone or vehicle in anxiety model caves	16 (8+8) wistar rats + 8 sprague-dawley rats	Agomelatine 10, 20, 50, 75, 100, 125 mg/kg; melatonin 10, 20, 50 and 75 mg/kg; diazepam 2.5 and 5.0 mg/kg; buspirone 2.5 mg/kg; vehicle (1% hydroxyhethylcellulose) 1 ml/kg	Agomelatine displayed a pattern of anxiolytic activity resembling that of diazepam (but without sedative properties) and buspirone, while melatonin was far less active
**Table 1 part b**						
Loiseau *et al*. (2005)	Animal model (murine)	Animal model of depression (impulsive-related behavior)	Rats were trained in a T-maze and allowed to choose between two magnitudes of reward: immediate but small reward (two pellets) vs. 25-s delayed but large reward (ten pellets); rats behavior was examined after the administration of agomelatine, GR205171 (substance P receptor antagonist) and melatonin, in comparison to positive controls clomipramine and fluvoxamine	10 rats per group, wistar rats	Agomelatine 10 and 30 mg/kg; GR205171 10 and 30 mg/kg; melatonin 3 and 10 mg/kg; clomipramine 8 mg/kg; fluvoxamine 4 mg/kg; vehicle (tween 80)	As clomipramine and fluvoxamine, agomelatine and GR205171 significantly increased the number of choices of the large-but-delayed reward. These results suggest that agomelatine enhances rats tolerance to delay gratification, an effect which may reflect its ability to improve impulse control
Barden *et al*. (2005)	Animal model (murine)	Transgenic mouse model of the neuroendocrine characteristics of depression (low glucocorticoid receptor functioning)	Behavioral changes (porsolt forced swim test and elevated plus maze test), body temperature and ACTH and corticosterone levels were analyzed in transgenic mice after the administration of agomelatine, melatonin, desipramine or vehicle	185 transgenic mice (bearing the glucocorticoid receptor antisense construct) and 115 non-transgenic mice (controls)	Agomelatine 10 mg/kg; melatonin 10 mg/kg; desipramine 10 mg/kg; vehicle (hydroxyethylcellulose 1%)	Agomelatine was effective in reversing the transgenic mouse behavioral changes, as well as desipramine or melatonin; agomelatine, but not imipramine, accelerated resynchronization of transgenic mouse circadian cycles of temperature and activity (this effect of agomelatine was more rapid than that of melatonin); no changes on concentrations of corticosterone and ACTH
Millan *et al*. (2005)	Animal model (murine)	6 different stress-induction models	Anxiolytic profile of agomelatine was compared with clorazepate and SB243,213 (selective 5-HT2c receptor antagonist) through a combined neurochemical and behavioral approach	Wistar rats and NMRI mice, unspecified number	Agomelatine, 0.63 to 80 mg/kg vs. melatonin (2.5-160 mg/kg) vs. SB243,213 (0.01-40 mg/kg) vs. clorazepate (0.63-40 mg/kg) vs. vehicle	The anxiolytic profile of agomelatine differs from that of benzodiazepines from which it may also be distinguished by its contrasting influence on cortico-limbic monoaminergic pathways
Tuma *et al*. (2005)	Animal model (murine)	Animal model of anxiety (social defeat)	SCN-lesioned and non lesioned rats, subjected at a social defeat by an aggressive opponent	Unspecified n.	Agomelatine – variable doses	Agomelatine caused a clear reduction of the social defeat induced behavioral consequences only in the non lesioned rats, indicating that the anxiolytic -like action of agomelatine requires the integrity of the SCN
Bourin *et al*., (2004)	Animal model (murine)	Animal model of depression	Forced swimming-test (FTS) in differently treated rodents (rats and mice).	10 mices per group (4) and 6 rats per group (4)	Melatonin (4, 8, 16, 32, 64 mg/Kg) vs imipramine (64 mg/Kg, 8mg/Kg), fluoxetine (16mg/kg).	Antidepressant efficacy shown
**Table 1 part c**						
Hanoun *et al*. (2004)	Animal model (murine)	Animal model of depression	Binding/electrophysiological, comparative study on the 5-HT_1A_ modulation by SSRI (fluoxetine) vs agomelatine	13+(5+5)+(5+5) rats	Agomelatine 10mg/kg/day vs melatonin (10mg/kg/day) and agomelatine (50mg/Kg/day) vs fluoxetine (5mg/kg.day)	Agomelatine Antidepressant effect is not related to 5-HT_1A_ modulation (as expected for other AD classes)
Chagraoui *et al*. (2003)	Animal model (murine)	Dose-dependent effects of agomelatine in preventing penile erections in rats induced by 5-HT2c receptor agonists	Penile erections were measured in rats after the injection of the 5-HT2c agonists ( mCPP and Ro 60-0175), with or without agomelatine (and other melatonin derivates) pretreatment	80-160 wistar rats	Agomelatine 1.25-40 mg/kg; melatonin 1.25-40 mg/kg; mCPP 0.75 mg/kg; Ro 60-0175 2.5 mg/kg	Agomelatine, but not melatonin, dose-dependently decreased mCPP- and Ro 60-0175- induced penile erections in rats, most probably due to its 5-HT_2c_ receptor antagonism
Millan *et al*. (2003)	Animal model (murine)	Binding affinities, in vivo, evaluation study	Antagonism at 5-HT_2C_ receptors and blockade was evaluated with *in vivo* receptor binding assays and measures	Wistar rats (unspecified number)	Agomelatine, 0.16 to 80 mg/kg vs. melatonin (2.5-40 mg/kg)	In contrast to melatonin, agomelatine behaves as an antagonist at 5-HT2c receptors, increasing extracellular levels of DA and NA in FCX and accelerating the firing rate of adrenergic cell bodies in the locus coeruleus; hence enhancing the activity of fronto-cortical DA-ergic and adrenergic pathways
Papp *et al*. (2003)	Animal model (murine)	Animal model of depression (chronic mild stress)	Sucrose test in rats subjected to the chronic stress procedure (food and water deprivation; 45° cage tilt; intermittent illumination; soiled cage; low intensity stroboscopic illumination) after evening or morning administration of agomelatine, melatonin, imipramine, fluoxetine or vehicle	336 wistar rats	Agomelatine 10 and 50 mg/kg; melatonin 10 and 50mg/kg; imipramine 10 mg/kg; fluoxetine 10 mg/kg; vehicle (1% hydroxyethylcellulose) 1 ml/kg	Antidepressant-like activity of agomelatine was shown to be independent on the time of drug administration; the efficacy of agomelatine is comparable to that of imipramine and fluoxetine, but greater than melatonin’s one
Tuma *et al*., (2001)	Animal model (murine)	Continuous dark exposure with consequent phase shift in circadian pacemaker	The free-running body temperature and activity rhythms were studied by gradual phase advances of the start of activity phase	Rats (various sp.)	Agomelatine up to 20mg/kg/day	Agomelatine treatment resulted in prolonged overstimulation of melatonin receptors, attenuating the effects of light on the circadian timing system.
Van Reeth *et al*., (2001)	Animal model (murine)	Age-related changes in circadian response to environmental stimuls	Young and older hamsters fed with melatonin or its agonist agomelatine	12+14 young (8 wk old) and 12+14 old (10 mo) hamsters	Variable doses	6 of 7 young hamsters fed with agomelatine showed large phase advance vs only 2 (of 8) old controls
**Table 1 part d**						
Chu *et al*. (2000)	Animal model (murine)	Post-mortem evaluation of DA-ergic and PRL act. on TIDA neurons	Simultaneous determination of serum PRL and DOPAC levels in the median eminence (as indices for TIDA neuronal activity) in ovariectomized, estrogen-treated rats after time-dependent injections of melatonin, agomelatine, S-20928 or vehicle.	50 sprague-dawley rats	Agomelatine 1 mg/kg; melatonin 0.01-1 mg/kg; S-20928 1 mg/kg	Melatonin and agomelatine exerts an inhibitory effect on PRL secretion by stimulating the TIDA neurons	
Weibel *et al*. (2000)	Animal model (murine)	Circadian resynchronization in old hamsters after abrupt shifts in the light-dark cycle	Running-wheel activity was monitored in two groups of hamsters (agomelatine-treated vs. control) subjected to an abrupt 8 h advance shift in the light-dark cycle (“jet-lag” paradigm)	24 golden hamsters	Agomelatine 20 mg/kg	Agomelatine accelerated by 25% resynchronization of the circadian rhythm in hamsters to the new light-dark cycle	
Pitrosky *et al*. (1999)	Animal model (murine)	Organization of rat circadian rhythms during daily infusion of melatonin or agomelatine	Running-wheel activity, body temperature and general activity were monitored in rats in constant darkness during a period of daily infusions of melatonin or agomelatine for 1, 8 or 16 h	110 long-evans rats	Agomelatine 50 and 100 microg/h; melatonin 50 and 100 microg/h	Agomelatine and melatonin entrained the free-running circadian rhythms of rats	
Ying *et al*., (1998)	Animal model (murine)	*In vivo* electrophysiological monitoring of SCN activity	Male Syrian hamsters were chronically exposed to melatonin and agomelatine to assess wherever this could influence later response of SCN receptorial activity	50 caves in four groups: vehicle (2), melatonin and agomelatine	Agomelatine at 1mg/kg	Chronic SCN melatonin receptor exposure to agomelatine does not alter their effects on suprachiasmatic nucleus neurons	
Masson-pevet *et al*. (1998)	Animal model (murine)	Binding studies by quantitative autoradiography	Record of the effects of agomelatine, S-20928 and melatonin on melatonin receptors in the rat pars tuberalis	Unspecified n.	Agomelatine – variable doses	Agomelatine was able to down-regulate melatonin receptors in the rat pars tuberalis	
Mauriño *et al*. (1998)	Human blood mononuclear cells	Binding studies	Binding studies and cytokine determinations on human blood mononuclear cells after the administration of agomelatine (specific membrane receptor agonist), CGP 52608 (RZR/ROR nuclear receptor agonist) and melatonin (membrane and nuclear receptors agonist)	Unspecified n.	Agomelatine–variable doses	While melatonin and CGP 52608 increased IL2 and IL6 production (due to their activity on nuclear receptor), agomelatine did not stimulate cytokine production	
Redman and Francis (1998)	Animal model (murine)	Pineal gland taxotomy comparative models	Locomotor activity and body temperature rhythms were examined prior and after the injection of agomelatine or vehicle to assess the role of the suprachiasmatic nuclei (SCN) and of the pineal gland in the entrainment of circadian rhythms by agomelatine	52 long-evans hooded rats	Agomelatine 1-10 mg/kg; vehicle (DMSO 50%)	Entrainment of circadian rhythms by agomelatine requires intact suprachiasmatic nuclei but not the pineal gland	
Table 1 part e						
Van Reeth *et al*., (1998)	Animal model (murine)	Motor/ general activity reduction (depression) by dark prolonged exposure	“jet-lag” paradigms involving phase shifts in light-dark (LD) cycle, induced to investigate the effects of S-20098 on the circadian clock of diurnal rodents.	Male Arvichantis rodents (unspecified number)	Agomelatine (20mg/day/kg) on the day of shift and also on subsequent 6h or 8h shift paradigms.	In each condition, agomelatine accelerated by about 30% resynchronization to the new LD cycle.
Wiley (1998)	Animal model (murine and monkeys)	Discrimination-reinforcement test in rats and monkeys	10 adult Sprague-Dawley rats and 4 adult rhesus monkeys (Macaca mulatta) were trained to discriminate diazepam/ agomelatine and methohexital/agomelatine respectively	10 adult rats and 4 adult monkeys	Diazepam 2.5mg/kg and methohexital 0.1mg/kg	Subjects preferred diazepam or non-melatonergic drugs to agomelatine, possibly indicating a non-addictive feature of the latter
Tenn and Niles (1997)	Animal model (murine)	Modulation of rat dopaminergic activity by agomelatine	Apomorphine-induced turning behavior was monitored in 6-hydroxydopamine lesioned rats, after administration of agomelatine, agomelatine+flumazenil, agomelatine+bicuculline or vehicle; competition binding assays (binding affinities of agomelatine compared to clonazepam, diazepam, and melatonin at benzodiazepine/GABAA receptors in the striatum)	34 sprague-dawley rats	Agomelatine 5 mg/kg; flumazenil 10 mg/kg; bicuculline 5nmol; apomorphine 0.25 mg/kg	Agomelatine inhibited apomorphine-induced turning In lesioned rats, showing an antidopaminergic effect (co-administration of flumazenil or bicuculline blocked this effects); agomelatine also inhibited [3H]diazepam binding striatal membrane; so the antidopaminergic action of agomelatine was mediated by BZ/GABAA receptors in the striatum
Van Reeth *et al*., (1997)	Animal model (murine)	Motor/ general activity reduction (depression-like) by dark prolonged exposure	Sleep phase in mice and Syrian hamsters was investigated by Phase-Response-Curve (PRC) observation. Motor activity was evaluated by daily wheel revolutions record.	45 (dark exposure and treatment varied among selected individuals)	Agomelatine (10mg/kg/day up to 20/25 mg/Kg/day, respectively in mice and hamsters)	Agomelatine showed dose-dependent phase shifting effects on all the used rodents, also improving motor activity.
Martinet *et al*. (1996)	Animal model (murine)	Wheel-running activity monitoring in rats free-running in constant darkness	Dose- and concentration- dependent effects of agomelatine on entraining circadian rhythms of rats	106 Long-evans rats	Agomelatine 0.5-10 mg/kg; melatonin 8 mg/kg; ipsapirone 8 mg/kg; vehicle (hydroxiethylcellulose 1% in H_2_O_2_)	Agomelatine was as effective as melatonin to entrain free-running rhythms; agomelatine showed dose-depended response from 2.5 to 10.0 mg/kg, and also a clear relation between entrainment and plasma concentration
Grassi-Zucconi *et al*. (1995)	Animal model (murine)	Animal model of dysfunction of the sleep regulatory mechanisms	EEG recording in trypanosome- infected rats after administration of agomelatine, melatonin or vehicle (trypanosome infection in the rat reduced selectively the length of synchronized sleeps episodes)	36 infected wistar rats and 17 non-infected wistar rats	Agomelatine 3 mg/kg; melatonin 3 mg/kg; vehicle (DMSO)	Agomelatine and melatonin restored a normal sleep pattern during the infection, increasing the length of synchronized sleep episodes
Table 1 part f						
Redman *et al*. (1995)	Animal model (murine)	Circadian resynchronization in rats after shifts in the light-dark cycle	Using 8h phase advance paradigm, the effects of daily-injections of agomelatine on the rat activity rhythms were compared with those of melatonin	Unspecified n.	Agomelatine 1.0-100 mg/kg	Agomelatine altered the direction of re-entrainment of rat activity rhythms in the same manner as melatonin; the effect was dose-dependent, with 100% of rats responding at a dose of 100 mg/kg
Tobler *et al*. (1994)	Animal model (murine)	Reduced vigilance model in rats	The vigilance states, electroencephalogram power spectra (0.25-25.0 Hz), and cortical temperature were monitored in rats after the administration of agomelatine, melatonin or vehicle	8 rats	Agomelatine 3 mg/kg; melatonin 3 mg/kg	Agomelatine and melatonin reduced the power density in non-rapid eye movement sleep in the low frequency range (1-8 Hz) but did not affect the vigilance states and brain temperature
Armstrong *et al*. (1993)	Animal model (murine)	Animal model of delayed sleep-phase syndrome	Rats were held for 3 months in constant darkness; when they returned in a light-dark cycle, the onset of activity lags behind the onset of darkness by 3-4 h; rats activities was monitored after injected with agomelatine, melatonin or vehicle	24 long-evans rats	Agomelatine 1 and 3 mg/kg; melatonin 1 mg/kg; vehicle (dimethylsulphoxide 50%)	Agomelatine and melatonin phase advanced the onset activity toward the onset of darkness

**Table 2:** Agomelatine in Mood and Anxiety Conditions and More. Current Literature Evidences in Clinical Human Studies.

Author, Date	Disorder	Method	*N* Patients	Dose/Range	Results	Side-Effects
**Table 2 part a**						
Goodwin *et al*., (2009)	MDD	24-week, placebo-controlled, randomized clinical trial	339 (165 on agomelatine, 174 on placebo)	25 or 50 mg/day	Agomelatine was efficacious in preventing major depressive episode (MDE) recurrence while withdrawal syndrome was almost absent (placebo comparable profile)	
Kennedy *et al*., (2008)	MDD	12 weeks double-blind, multicenter study, comparison of sexual functioning, antidepressant efficacy and tolerability between agomelatine and venlafaxine	137(agomelatine) and 140 (venlafaxine RP)	50 mg/day ago, tritated to a target dose of 150 mg/day venlafaxine	Agomelatine showed antidepressant efficacy and a superior sexual side effect profile vs. venlafaxine XR	
Montejo *et al*., 2008	Healthy volunteers	8 weeks placebo-controlled study using PRSEXDQ-SALSEX scale to study sexual acceptability of ago compared with paroxetine	92	25-50 mg/die ago 20 mg/die paroxetine	Sexual Dysfunction was significantly lower in ago group than in paroxetine group	None
Stein *et al*. (2008)	GAD	12 weeks randomized, double-blind, placebo-controlled trial	121	25-50 mg/die ago	Significant superiority of ago 25 to 50 as compared with placebo; clinical response, symptoms of insomnia and improvement in associated disability, were consistent with the efficacy of ago.	Any relevant
Calabrese *et al*., (2007)	Depressed Bipolar I co-medicated with lithium or valpromide	Open-label for a minimum of 6 weeks followed by an optional extension of up to an additional 46 weeks	14(lithium) 7(valpromide)	25 mg/day agomelatine	Effectiveness of agomelatine	Any relevant
Lemoine *et al*., (2007)	MDD	Placebo-controlled RCT: 2 arms, venlafaxine vs agomelatine	332	25-50mg/day ago or 75-150mg/day venlafaxine (variable dose)	The 6 weeks antidepressant effect of agomelatine was similar to those of venlafaxine. Sleep quality (measured by LSEQ) was subjectively better among patients treated with agomelatine.	Few with venlafaxine (dizziness, nausea, vomiting, tremor etc…), almost any with ago
Lopes *et al*., (2007)	Non-REM sleep instability in MDD	Single-blinded	15+15	25mg/day	Agomelatine improved NREM sleep phases	Out of study aims
Montgomery and Kasper (2007)	Severe Depression	Pooled analysis of 3 positive placebo-controlled studies	357 (agomelatine) and 360 (placebo)	25-50mg/day	Clearly effective	Any relevant
Olié and Kasper, (2007)	Moderate to severe MDD	6 weeks, double-blind, placebo-controlled, parallel randomized, group study (variably doses)	238	25mg/day (augmented to 50mg/ day after 2weeks of non-response)	Depressed and sleep items improved in moderate and severe depressed patients	Placebo comparable frequency and severity
Pjrek *et al*., (2007)	SAD	14 weeks open study	37	25 mg/day	Large percentage of patients experiencing sustained remission during the 14 weeks of this study	Only one adverse event: mild fatigue
Quera Salva *et al*., (2007)	MDD	Open-label, polysomnography (PSG), quantitative EEG	15	25mg/day agomelatine for 6 weeks	Sleep efficiency increased and intra-sleep awakening progressively decreased	Any relevant
Kennedy and Emsley, (2006)	Current (monopolar) MDE	Placebo-controlled 6 weeks RCT	212	25-50mg/day ago	Both doses resulted to be well tolerated and effective also in severe cases (50mg/day)	Any relevant
**Table 2 part b**						
Montgomery *et al*., (2004)	MDD	RCT: patients treated for 12 weeks with paroxetine 20mg/day vs patients treated with ago 25mg/day for 12 weeks, were abruptly discontinued to placebo or continued their drug for 2 more weeks.	192	20mg/day (paroxetine) or 25mg/day (ago)	Patients treated for 12 weeks with agomelatine and continued to 2 weeks on the same drug, showed similar discontinuation symptoms to those “continued” to placebo while the paroxetine discontinued group experienced more.	
Loo *et al*., (2003)	DSM-III-R diagnosed MDD	RCT	14 inpatients+14 outpatients	5-100mg/day ago	Acceptability, efficacy were confirmed both at 5 and 100mg/day doses. 5mg regimen offered best clinical outcome while 100mg/day resulted in greater side effects frequency and drop-outs	Any relevant
Loo *et al*., (2002)	MDD	8 weeks double-blind, placebo-controlled dose range study; paroxetine was used as the study validator	711	1 mg/die or 5 mg/die or 25 mg/die ago	Ago 25 mg/die is statistically more effective than placebo in MDD and alleviates the anxiety associated with depression.	Any relevant
Cajochen *et al*., (1997)	Healthy volunteers	Cross-over design, comparison of acute administration of melatonin vs agomelatine 5h prior to bed time. Sleep structure and EEG evaluations.	8 young male students (23-32 years)	5-100mg/day (melatonin/ ago)	A single early dose of melatonin or agomelatine increases REM sleep propensity and advances sleep termination without affecting NREM duration.	None
Kräuchi *et al*., (1997)	Healthy volunteers	Double-blind, placebo-controlled crossover. Administration of melatonin, agomelatine and placebo was compared with dim-light onset, distal and core body temperature registrations.	8	5mg/day (melatonin), 5 or 100mg/day (agomelatine)	Dose-dependent administration of melatonin or agomelatine resulted in earlier regulation of the endogenous circadian nocturnal decline in core body temperature and circadian phase advance.	Out of study aims

**Table 3:** Comparison of SSRIs Efficacy and Tolerability vs. Agomelatine

	SSRIs	Agomelatine
**GI disturbances**	x [a]	0 [c]
**Long-term weight gain**	x/xx [a]	0 [c]
**Daytime sleepiness**	x/xx [a]	0 [c]
**Sexual dysfunction (may be dose-related)**	xx/xxx [a]	0 [c][d]
**Discontinuation symptoms**	x/xx [a]	0/x [e]
**Efficacy on more severe depression**	“questioned” [b]	Preliminary [f]observations reported agomelatine to be efficacious as long-term RCT evidences showed its efficacy in the prevention of major depressive episode recurrence [g]

[a] Masand and Gupta, 1999

[b] Anderson, 2000; Sonawalla and Fava, 2001; Vestergaard *et al*., 1993

[c] Hindmarch *et al*., 2000; Judge *et al*., 2002; Michelson *et al*., 2000; Rosenbaum *et al*., 1998; Montgomery *et al*., 2004

[d] 100 mg/day regimen of agomelatine were reported to be associated with possible side effects (Loo *et al*., 2003)

[e] Discontinuation cases may be related to a non satisfactory antidepressant response when a low-dose, monotherapy regimen, is established and may be comparable to placebo-related ones (Goodwin *et al*., 2009)

[f] Olié and Kasper, 2007

[g] Goodwin *et al*., 2009

*Note*: “0” should be clinically considered as a “placebo-comparable” profile in most of the cases
